# Forging the microbiome to help us live long and prosper

**DOI:** 10.1371/journal.pbio.3002087

**Published:** 2023-04-05

**Authors:** Rachel R. Rock, Peter J. Turnbaugh

**Affiliations:** 1 Department of Microbiology and Immunology, G.W. Hooper Research Foundation, University of California, San Francisco, California, United States of America; 2 Chan Zuckerberg Biohub–San Francisco, San Francisco, California, United States of America

## Abstract

Aging is often accompanied by an increased risk of an array of diseases spanning the cardiovascular, nervous, and immune systems, among others. Despite remarkable progress in understanding the cellular and molecular mechanisms involved in aging, the role of the microbiome remains understudied. In this Essay, we highlight recent progress towards understanding if and how the microbiome contributes to aging and age-associated diseases. Furthermore, we discuss the need to consider sexually dimorphic phenotypes in the context of aging and the microbiome. We also highlight the broad implications for this emerging area of interdisciplinary research to address long-standing questions about host–microbiome interactions across the life span.

## Abstract 2

Aging and the microbiome are linked to similar pathological and molecular phenotypes, yet these two fields rarely intersect. This Essay highlights recent progress towards clarifying the microbiome’s role in aging and the need to account for sex and other confounding factors.

## Introduction

Age represents the primary risk factor for some of the most prevalent diseases of high-income countries, including cancer, cardiovascular disease, and neurodegeneration [1]. Increased age is also associated with the risk and/or severity of numerous other diseases, including multiple sclerosis [2] and type 2 diabetes [3]. Defining mechanisms that contribute to health and disease in aging will therefore not only expand our knowledge of this universal process but also pave the way for interventions enabling us to optimize health in aging individuals.

The trillions of microorganisms found in and on the human body (the microbiota) offer tremendous potential in understanding aging. The microbiome (the aggregate genetic content of the microbiota) exceeds the human genome by multiple orders of magnitude [4]. Microorganisms colonize numerous sites in and on the body, with the greatest extent of colonization occurring within the gastrointestinal (GI) tract [4]. Extensive and rigorous prior research has emphasized the key role that the gut microbiota has in host health and disease, including contributions to diseases associated with aging such as cancer [5–7], Parkinson’s disease [8,9], obesity [10,11], and type 2 diabetes [12]. Yet, despite remarkable progress in understanding the cellular and molecular mechanisms through which the microbiome contributes to individual diseases linked to aging, the net effects of the microbiome for the aging process or the potential for manipulating the microbiome to promote healthy aging remain unclear.

This work is further complicated by the many demographic factors that contribute to aging and age-related phenotypes. For example, sex is an important factor in aging [13]. Women significantly outlive men in nearly all human populations around the world [14], and most of the World Health Organization’s common age-associated causes of death are sexually dimorphic [15]. The mechanisms responsible for these sexually dimorphic phenotypes remain poorly understood, emphasizing the need to better understand if and how differences in the microbiomes of male and female animal models influence their health and longevity as well as the conservation of these phenomena in humans.

In this Essay, we discuss the emerging literature indicating that the human microbiome is altered in aging individuals and that the microbiome impacts longevity in model organisms. We highlight recent studies in humans and model organisms that implicate the microbiome in multiple age-associated diseases, focusing on cancer, obesity, type 2 diabetes, and Parkinson’s disease. Then, we explain why biological sex is a key gap in understanding how the microbiome shapes aging. Together, these discussions emphasize the broad impact of the microbiome across the life span and the potential for rapid new discoveries in this interdisciplinary area.

### The microbiome and aging

#### The human microbiome is altered in elderly adults

The overall association between the human microbiome and age is strong enough that it is possible to predict biological age with striking precision with the microbiome. An initial proof-of-concept was demonstrated in early life, in which a “microbiota maturity index” established in healthy individuals was delayed in the context of malnutrition [16]. More recently, machine learning tools have enabled the accurate prediction of age in adults from distal gut metagenomic data with a mean absolute error of 6 to 8 years [17,18]. The composition of the microbiota found in other body habitats, including the skin and oral cavity, is also linked to age [19]. The skin microbiota has even been used postmortem to date bodies [20,21], emphasizing that the temporal relationships with the human microbiota encompass the entire life span and shortly thereafter. Continued progress in this area has clear implications for forensics, enabling new approaches to identifying suspects [22] and potentially even their age. Microbiome signatures have also been associated with survival in the elderly [23], further underscoring the importance of understanding how the microbiome is altered in aging.

**Fig 1:**
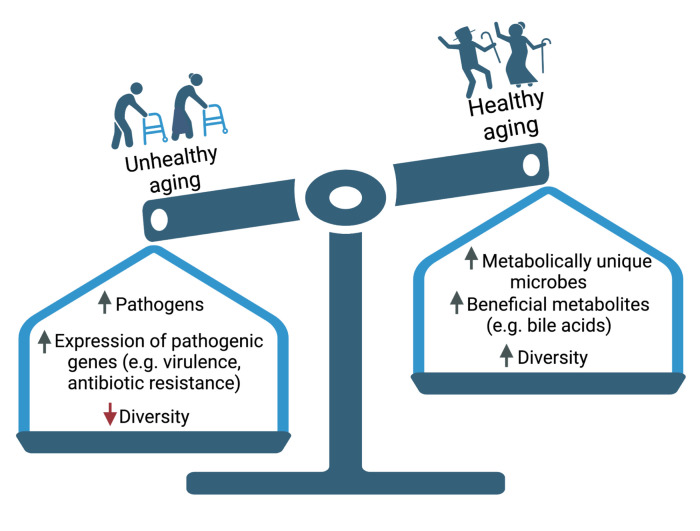
Lessons from the extremes of aging: Healthy centenarians and frail elderly. Aging can be considered as opposite sides of a balance tugged by the weights of various microbial factors, discussed in detail in this Essay. Created with BioRender.com.

Work on centenarians (individuals aged 100+ years) has provided valuable insights into components of the microbiome that may promote healthy aging [24] (Fig 1). Centenarians exhibit a higher bacterial diversity than younger individuals and are enriched for the bacterial genera *Alistipes*, *Parabacteroides*, and *Clostridium*. Consistent with these taxonomic shifts, multiple microbial metabolites are also enriched in centenarians, including anti-inflammatory bile acids produced by gut bacteria [24]. Follow-up studies testing the causal role of the specific bacterial species, genes, and metabolites in promoting healthy aging are needed; however, these data clearly demonstrate that individuals at the extremes of longevity harbor distinctive microbial taxa and metabolic end-products.

Frailty (the condition of being weak or vulnerable to biological stressors) has also been linked to interindividual differences in the human gut microbiome [25,26] (Fig 1). Frail elderly individuals have decreased gut bacterial diversity relative to less frail individuals after adjusting for age [25]. Longitudinal analysis of community-dwelling and skilled nursing facility-dwelling older adults has revealed frailty-associated differences in the skin, oral, and gut microbiota [26]. Multiple potentially pathogenic bacterial species were observed on the skin of frail older adults, together with a vast repository of antibiotic resistance genes [26]. As in centenarians, the causal role of the microbiota in driving frailty remains to be established, especially given the many confounding factors that could potentially explain these frailty-associated differences in the human microbiota.

Age is associated with multiple aspects of lifestyle and changes in host biology that could feasibly explain many or all of the observed differences in the human microbiota. Aging is accompanied by impaired host immunity [27], which could lead to an expansion of microorganisms that were formerly held in check by the immune system, possibly explaining the enrichment of potential bacterial pathogens in frail elderly individuals [26]. Diet is also an obvious confounding factor, as the more restricted diet of nursing home residents may be a key driver of changes in the gut microbiota in some elderly individuals [28]. Gut motility also generally slows with age [29], which could have downstream consequences for the gut microbiota [30]. Finally, social determinants of health in aging such as solitary living [31], increased likelihood of residential care [32], reduced mobility [33,34], and loss of interpersonal relationships [35,36] could all potentially influence the microbiome. Given the numerous factors that could have a role, a recent study took a more integrative approach, demonstrating an association between the gut microbiome and overall life history, which encompasses information about medications, physical activity, diet, and blood markers [37]. Thus, microbiome shifts with respect to age appear to be driven by the net effects of numerous host and environmental factors.

These results emphasize that the human microbiome is an important but understudied aspect of the aging process. Given the complexity of this microbial ecosystem, disentangling causal relationships is intractable in humans, motivating the emerging work in model organisms that we discuss in the following section.

#### The microbiome impacts longevity across model organisms

Research in germ-free (GF) model organisms has provided strong support for a causal role of the microbiome in determining host life span, including studies in worms, flies, fish, and mice. Considered together, the results of the studies discussed below imply that the human microbiome also has a causal role in life span; however, the direct “reverse translation” of the specific aspects of the human microbiome associated with aging to these model organisms remain to be explored.

Research across multiple model systems suggest that exposure to the microbiome in early life is beneficial in extending life span. This is most extreme in *Danio rerio*, which do not reach maturity under GF conditions due to an epidermal degeneration phenotype, likely driven by inadequate nutrition [38]. Similarly, bacterial colonization during embryonic development extends the life span of *Drosophila melanogaster* [39]. However, these results conflict with data from GF *Caenorhabditis elegans* [40], GF mice [41,42], and GF rats [42,43], which all live longer than conventionally raised (CONV-R) control animals. Thus, the potential benefits of microbial colonization in early life may be outweighed by detrimental effects later in life.

Consistent with this hypothesis, the microbiome can decrease life span in older animals. In *C*. *elegans*, GI accumulation of *Escherichia coli* contributes to age-related death [44]. Removal of GF *D*. *melanogaster* from sterile conditions reduces life span in adults [39]. More recently, the detrimental effects of the microbiome in aging animals has been studied using the African turquoise killifish [45]. Middle-aged (9.5-week-old) killifish treated with antibiotics outlived untreated fish, suggesting that the microbiota impairs life span in older killifish. Remarkably, inoculation with the GI microbiota of 6-week-old killifish significantly increased the life span of middle-aged killifish groups [45].

These findings are also relevant to mammals. Work in 2 mouse models of progeria (a human premature aging syndrome) supports the potential for microbiome-based interventions to extend life span [46]. The gut microbiota was altered in prematurely aging mice, including a significant decrease in *Akkermansia muciniphila* in the *Lmna*^G609G/G609G^ model, which harbors the nuclear envelope lamin A/C point mutation responsible for the most common human progeria syndrome. As in killifish [45], fecal microbiota transplantation (FMT) from wild-type mice significantly increased the life span of transgenic prematurely aging recipient mice. Even more excitingly, the Verrucomicrobium *A*. *muciniphila*, a common member of the human gut microbiota, was sufficient to extend life span in the mice [46]. These results provide a major step towards identifying the cellular and molecular mechanisms responsible for microbiota-dependent changes in life span as well as an important step towards the potential translation of these results to humans.

One mechanism supported by results in multiple model organisms is that the microbiome may decrease life span by increasing the accessibility of dietary nutrients. Thus, differences in the microbiome may counteract or even compound the effects of caloric restriction, which extends life span in multiple species [47]. This area of study will benefit from the already extensive literature focused on the role of the gut microbiome in nutrition [48]. Briefly, the microbiome is critical for the digestion of plant polysaccharides [49], the absorption of lipids [50], and the uptake of amino acids [51]. Microbial colonization can also activate multiple pathways inhibited by caloric restriction (which extends life span), including insulin-like growth factor 1 [52] and AMP-activated protein kinase [53]. Notably, GF mice lose their life span advantage over CONV-R mice when calorie restricted [54]. Furthermore, recent studies in humans and mouse models have revealed that caloric restriction can perturb the human gut microbiome in a manner that promotes weight loss [55]. Extensive data have also implicated the microbiome in malnutrition [56]. More work is needed to untangle these complex interactions between diet and microbiome and their long-term implications for host health and longevity.

### The microbiome and age-associated diseases

**Fig 2:**
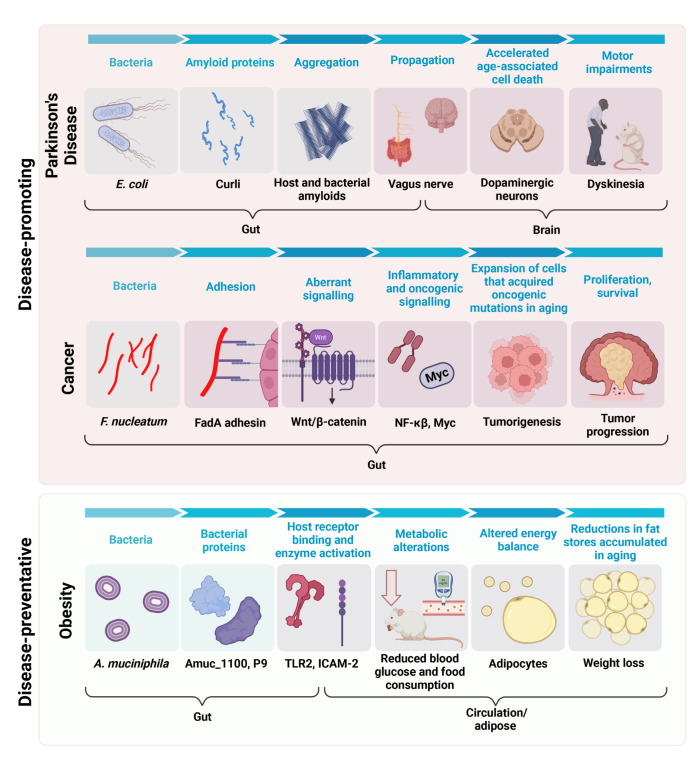
The microbiome influences age-associated disease. Diagram summarizing some of the mechanisms through which the microbiome has been implicated in 3 distinct age-associated diseases. We propose that the net effect of all these pathways shapes life span by dictating the risk and treatment of disease. Amuc_1100, *A*. *muciniphilia* protein 1100; FAD-A, *Fusobacterium* adhesin A; ICAM-2, intercellular adhesion molecule 2; NF-κB, nuclear factor-κB; P9, carboxyl-terminal protease; TLR2, Toll-like receptor 2. Created with BioRender.com.

The data discussed in the previous section emphasize that the microbiome could influence longevity through shaping the risk and treatment of disease. Recent investigation supports that host age contributes to differences between disease-associated microbiomes and those of healthy individuals [57]. Given the vast literature spanning multiple disease areas, we opted to focus the following sections on 3 age-associated disease areas: cancer, metabolic disease (obesity and type 2 diabetes), and Parkinson’s disease (Fig 2). The studies discussed here highlight the potential to pair mechanistic and translational microbiome research and the generalizability of these approaches to other age-associated diseases.

#### The microbiome influences cancer risk and treatment outcomes

Cancer is associated with age, with under 25 cases per 100,000 under the age of 20, 350 cases per 100,000 among those aged 45 to 49, and over 1,000 cases per 100,000 in individuals 60 years and older [58]. The majority of cancer types, including breast [59], prostate [60], and colorectal [61], follow this trend. The causal role of the gut microbiome in cancer risk has been reviewed elsewhere [62], including seminal work on *Helicobacter pylori* [63]. More recently, comparisons of colorectal cancer tumors to adjacent, nonmalignant mucosa revealed a significant enrichment of *Fusobacterium nucleatum* [64]. Evidence for a causal role of *F*. *nucleatum* in colon cancer has come from mice, in which this bacterium activates signaling pathways that promote myeloid cell infiltration and expression of pro-inflammatory and oncogenic genes [65,66].

The entire microbiome, in addition to individual species such as *F*. *nucleatum*, can serve as a valuable biomarker for disease status. Using gut microbiome data as a screening tool improves colorectal adenoma prediction success by a factor of more than 50-fold [67]. The gut microbiome is also associated with cancers found in other organs, including the liver [68], prostate [69], and breast [70]. Furthermore, tumors found throughout the body often harbor detectable microorganisms, including both bacteria [71] and fungi [72], suggesting that the microbiome may have both local and systemic effects on tumor progression.

Work on cancer chemotherapy and immunotherapy has emphasized the broad role of the microbiome in shaping cancer treatment outcomes [73]. Remarkably, FMT from patients with melanoma who responded well to immunotherapy into other patients was followed by decreased tumor size in a subset of recipients [74,75]. These translational efforts were inspired by a series of elegant mechanistic studies highlighting how changes in the gut microbiome can alter host immunity and thus change responsiveness to immune checkpoint blockade [76,77]. In addition to immune interactions, the microbiome can also directly influence anticancer drugs by metabolizing them to downstream metabolites with increased [78,79] or decreased [79,80] activity. Selective inhibition of the bacterial enzyme that reactivates the anticancer drug irinotecan (β-glucuronidase) rescues GI toxicity [78], whereas high levels of expression of the bacterial *preTA* operon can interfere with the efficacy of capecitabine (an oral form of the anticancer drug 5-fluorouracil) [79]. Continued progress in understanding how the microbiome influences cancer risk, treatment, and survivorship has profound implications for addressing this devastating disease affecting the aging global population.

#### Reciprocal interactions between host metabolism and the microbiome

Obesity and type 2 diabetes are both associated with age [3,81] and have extensive ties to the microbiome (reviewed in-depth elsewhere [82]). In humans, consistent microbiome correlations with these conditions have been elusive due in part to the confounding effects of the diabetes medication metformin [83,84], gastric bypass surgery [85,86], and weight loss diets [55,87]. Differences between ethnic groups may also have a role: for example, in a United States–based cohort, obesity-associated differences in the gut microbiotas of white individuals were not detected in East Asian individuals [88]. Taken together, these results emphasize that the common medical interventions meant to ameliorate metabolic disease have profound impacts on the gut microbiome, which could also be relevant to the aging process. Furthermore, the specific microbial species, genes, and pathways involved may vary between individuals [89] and across cohorts [90], motivating efforts towards microbiome-informed precision nutrition and medicine.

Mechanistic work in model organisms has highlighted the numerous pathways through which the microbiome can influence phenotypes related to obesity and type 2 diabetes [82]. As discussed above, the microbiome can contribute to caloric intake by aiding in the digestion of otherwise inaccessible components of the diet [11], consistent with recent data in humans showing a significant decrease in dietary energy harvest following treatment with the antibiotic vancomycin [91]. In turn, the microbiome also impacts host energy expenditure, in part through changing host gene expression and enzymatic activity [53]. More recently, work on *A*. *muciniphila* has led to the identification of a bacterial protein that is sufficient to rescue mice from diet-induced obesity [92]. Additional research has identified a separate *A*. *muciniphila* protein sufficient to improve glucose tolerance and rescue a metabolic disease phenotype in mice [93]. These findings are consistent with data from humans supporting the safety and beneficial effects of pasteurized *A*. *muciniphila* [94]. Moving forward, it will be critical to see how the impact of the microbiome on host energetics changes in aging individuals, especially given the concomitant changes in dietary intake [95] and pharmaceutical use.

#### Connections between the gut and brain provide insight into neurological disease

The human microbiome may also have a causal role in the etiology and treatment of multiple neurological diseases whose risk and/or severity increase with age, including Alzheimer’s disease [96], multiple sclerosis [97], and Parkinson’s disease [98]. Here, we focus on Parkinson’s disease, given the recent advances towards understanding its relationship with the gut microbiome and the clear link to aging. More than 95% of Parkinson’s disease cases occur in individuals over the age of 50 years [99]; however, an aging population is insufficient to account for the rising incidence of Parkinson’s disease [100], implicating environmental factors like the microbiome. Furthermore, multiple lines of evidence implicate the GI tract in Parkinson’s disease: An early symptom to manifest is constipation [100]; the amyloid protein α-synuclein is found in the vagus nerve (which links the brain to the gut) before reaching the central nervous system [101]; and truncal vagotomy (removal of the vagus nerve at the gastroesophageal junction) is associated with a near 50% risk reduction of Parkinson’s disease [102,103]. Yet, despite these numerous links between the GI tract and Parkinson’s disease, the role of the microbiome has only recently come into focus.

Studies in mice have highlighted multiple mechanisms through which the gut microbiome communicates with the brain to impact Parkinson’s disease pathogenesis. The microbiota is altered in a mouse model of Parkinson’s disease wherein α-synuclein is overexpressed (the ASO model) [8]. Colonization of ASO GF mice [8], as well as an alternative mouse model of Parkinson’s disease [104], with the gut microbiota of affected mice or humans exacerbates brain pathology and motor dysfunction relative to controls. Disease can also be triggered by bacterial amyloids, as shown for the cell surface amyloid curli proteins made by *E*. *coli* [105]. Furthermore, a recent preprint demonstrates that gut bacteria can also influence the production of host amyloids, as bacterial nitrate reduction stimulates the intestinal aggregation of α-synuclein [106]. These results, combined with the growing amount of metagenomic data from patients with Parkinson’s disease and healthy individuals, suggest that multiple, distinct microbiome-dependent cellular and molecular mechanisms may combine to drive disease in patients with Parkinson’s disease [107].

The gut microbiome may also contribute to interindividual variations in Parkinson’s disease treatment outcomes. Parkinson’s disease treatment typically starts with the small molecule drug levodopa (L-dopa), which is converted in the central nervous system to dopamine, thus alleviating Parkinsonian symptoms resulting from neuronal dopamine depletion [108]. L-dopa is typically paired with carbidopa, a dehydroxylase inhibitor that reduces peripheral metabolism of the drug [109]. However, carbidopa does not inhibit the gut bacterial enzyme tyrosine decarboxylase (TyrDC) [110,111], which catalyzes the first step in a pathway for the gut bacterial metabolism of L-dopa to *m*-tyramine within the GI tract [111]. Instead, the compound (S)-α-fluoromethyltyrosine can be used to specifically inhibit bacterial TyrDC, leading to increased serum L-dopa in mice [111]. Notably, *tyrDC* levels increase over time in patients with Parkinson’s disease and are associated with GI adverse effects of treatment with multiple Parkinson’s disease medications [112]. TyrDC may be just one of multiple routes of gut bacterial metabolism; *Clostridium sporogenes* can also deaminate L-dopa [113]. More work is needed to understand the relative contributions of these and other pathways in model organisms and patients with Parkinson’s disease, as well as their downstream consequences for drug efficacy and adverse effect profiles. This concept could also be more broadly applied to other drugs used to treat neurological disease; for example, the Alzheimer’s disease medications galantamine and memantine, which are depleted by human gut bacterial isolates during in vitro growth [114].

### Sex is a key gap in understanding how the microbiome shapes aging

Aging is fundamentally distinct in men and women, with broad differences in life span, frailty, and age-related diseases [13]. Frailty in women persists throughout the life span, with disability peaking in later years [115]. Yet, women outlive men in nearly all human populations around the world [14]. These data hold even when adjusting for socioeconomic status, ethnicity, and education. Multiple molecular mechanisms contribute to sexual dimorphism in aging, including endocrine and host genetic differences. For example, while contradictory findings reporting more modest or even opposite effects exist [116,117], several reports suggest that in humans and mice, oophorectomy decreases health span and life span [118–120]. In contrast, while also a topic of debate [121], several lines of literature support that male gonads and hormones can negatively influence life span. For example, research in eunuchs suggests that castration increases longevity in men [122,123], and research in rodents has shown certain exogenous androgens to decrease life span [124]. Furthermore, gonad swapping experiments in mice support the conclusion that ovaries (and potentially their hormones) can significantly prolong life span [125].

Most diseases associated with aging are also sexually dimorphic, including the 3 disease areas highlighted above. Cancer incidence and survival are higher in women and girls [126], and numerous nonreproductive cancers are strongly sex biased in incidence, most notably endocrine cancers (female bias) and Kaposi sarcoma (male bias) [126]. Women are at increased risk of obesity compared with men [127] yet exhibit a comparable risk of type 2 diabetes [128]. Finally, neurodegenerative disease severity and risk track with sex: For example, Parkinson’s disease risk is higher in men, but women exhibit more severe disease [129].

An emerging literature has also begun to reveal links between sex and the microbiome in humans [130,131] and mice [132–134]. While the mechanisms responsible remain poorly understood, initial data point towards sex hormones as important mediators of this relationship. In humans, sex is associated with gut microbiota differences from puberty until the mean age of menopause, consistent with the hypothesis that sex hormones are an important driver of the observed differences [130].

In turn, the microbiome may also have an important role in controlling sex hormone levels. GF mice have altered sex hormone levels relative to CONV-R animals: GF males have lower testosterone and higher β-estradiol, whereas GF females have lower progesterone and β-estradiol [132,135,136]. Gut bacterial β-glucuronidases can reactivate estrogen glucuronides [137], consistent with data in humans linking antibiotics to decreased serum sex hormone concentrations and increased fecal excretion of sex hormone conjugates [138]. Furthermore, circulating sex hormone levels are associated with the diversity and composition of the gut microbiota [139].

While literature at the intersection of sex, microbiome, and aging remains sparse, some initial observations highlight the value of this line of inquiry. Work in GF mice suggests that the female longevity advantage requires the microbiota [41]. A seminal study in the nonobese diabetic model of type 1 diabetes revealed that sex differences in the microbiome can impact autoimmune disease [132]. Male CONV-R mice were protected from diabetes, but this difference was lost in GF males due to decreased testosterone. Remarkably, transplantation of the male-associated gut microbiota into female recipients was sufficient to protect from disease [132]. These effects are likely relevant beyond testosterone: A recent study of diet-induced obesity in mice suggested that estrogen-induced differences in the gut microbiome may protect from metabolic disease [140]. Moving forward, it will be critical to identify the mechanisms through which sex alters the microbiome and the downstream consequences for age-associated diseases and overall life span. In doing so, investigators ought to take important considerations in understanding how biological sex influences the microbiome’s effects on phenotypes of aging (Table 1).

**Table 1:** Accounting for biological sex in microbiome and aging research.

Observation	Limitation	Suggestion
Women significantly outlive men across populations	Cohorts become more female biased as participants age	Use sex as a factor for enrollment, using a priori power calculations to set minimums for enrollment of men and an acceptable male:female ratio
Hormonal contraceptive usage has increased globally	Hormonal contraceptives have unknown impacts on the microbiome	Collect data on hormonal contraceptive usage
Limited progress has been made towards inclusion of sex-specific analyses [141]	Many studies are underpowered to detect sex differences	Use both sexes in animal research, adequately power studies to detect sex differences, and report results using appropriate statistical methods
Aging rodents do not exhibit [142,143] the same decline in sex hormones observed in aging humans [144,145]	Wild-type rodents may not fully recapitulate all aspects of human aging	If relevant, use surgical, genetic, or pharmacological methods to model age-related depletion of circulating sex hormones
Research on sex in humans is confounded by gender	Associations in humans may reflect socioeconomic factors, not biology	Adjust for confounding variables in humans if possible and establish causality in animal models

## Conclusions

In this Essay, we discussed the emerging yet already compelling evidence supporting a role for the microbiome in aging and age-associated diseases. These findings have broad implications for biomedical science and other areas of biology. Microbiome researchers would do well to control for or otherwise account for age, sex, and other demographic variables in their studies, even if these variables do not represent the primary focus of their research program. In turn, researchers in the fields of aging and numerous age-associated disease areas should consider the potential role of the microbiome in their research; for example, by collecting exploratory samples for microbiome profiling, controlling for microbiome-associated variables like diet and cohousing, or using GF models [146]. Working together, this interdisciplinary research area is poised for rapid discovery and could address long-standing questions about the factors that control microbial community structure and function and the drivers of interindividual variations in age-related disease risk and treatment outcomes. Perhaps most importantly, it will be critical to avoid multiplying the hype in the microbiome and aging fields to prioritize rigorous, mechanistic, and experimentally tractable work aimed at understanding fundamental biological processes. The fountain of youth may be a long way off, but perhaps this line of research can still help us achieve more modest goals of living a bit longer and prospering a little bit more.

## Abbreviations

CONV-R: conventionally raised
FMT: fecal microbiota transplantation
GF: germ-free
GI: gastrointestinal
L-dopa: levodopa
TyrDC: tyrosine decarboxylase
